# Putting Patients First: Pragmatic Trials in Gynecologic Oncology

**DOI:** 10.3390/curroncol32030139

**Published:** 2025-02-27

**Authors:** Laura Hopkins, Mark Clemons, Karen Bemister, Chris Booth, Shannon Kadar, Paul Karanicolas, Julie Mulligan, Marie-France Savard, Ian Tannock, Alicia Tone, Helen MacKay

**Affiliations:** 1 Department of Oncology, University of Saskatchewan, Saskatoon, SK S7V 4H4, Canada; 2 Department of Oncology, University of Ottawa, Ottawa, ON K1H 8L6, Canada; mclemons@toh.ca (M.C.); msavard@toh.ca (M.-F.S.); 3 Patient Partners in Research, Ovarian Cancer Canada, Toronto, ON M2P 2A9, Canada; kbemister@rogers.com (K.B.);; 4 Department of Oncology, Queen’s University, Kingston, ON K7L 3N6, Canada; christopher.booth@kingstonhsc.ca; 5 Department of Surgery, University of Toronto, Toronto, ON M5S 3H2, Canada; paul.karanicolas@sunnybrook.ca; 6 Division of Medical Oncology, University of Toronto, Toronto, ON M5S 3H2, Canada; ian.tannock@uhn.ca; 7 Ovarian Cancer Canada, Toronto, ON M2P 2A9, Canada; atone@ovariancanada.org; 8 Division of Medical Oncology and Hematology, University of Toronto, Toronto, ON M4N 3M5, Canada; helen.mackay@sunnybrook.ca

**Keywords:** pragmatic clinical trials, gynecologic oncology, patient engagement, REaCT

## Abstract

In November 2024, the Society of Gynecologic Oncology of Canada hosted a 2-day, interdisciplinary Pragmatic Clinical Trials (PCTs) Workshop with the goal of launching an initiative to develop and promote PCTs within the Canadian gynecologic oncology research environment. The programme brought together multiple stakeholders, including patients with ovarian cancer, patient advocates, experts in PCTs, gynecologic oncologists, medical oncologists and clinical fellows. Foundational elements of pragmatism were emphasized in the context of the primary goal of PCTs, showing the real-world effectiveness of interventions in broad patient groups. Examples of how PCT outcomes can inform and influence clinical decision making and health policy were presented in the context of those outcomes that matter most to patients with cancer. The patients and patient advocates had the essential role of helping clinical investigators co-design PCT protocols to answer common, important, and practical questions that focus on outcomes that matter to patients. These endpoints included overall survival, quality of life and promotion of informed patient decision making. Tangible workshop outcomes included the development of several new proposals for PCTs inspirited and directed by the patient voice. Further educational initiatives to engage clinical gynecologic oncology investigators at all stages in their career are being planned.

## 1. Introduction

The number of patients with gynecologic malignancies, including ovarian, endometrial and cervical cancers, is increasing in Canada, with a combined 13,200 new cases and 4000 deaths estimated in 2024 by Canadian Cancer Statistics [1]. Of note, half of these deaths are due to ovarian cancer [2]. The physical, emotional and economic impacts of a gynecologic cancer diagnosis are substantial. Newer and frequently more toxic combination therapies are increasingly available, with the incorporation of molecularly targeted agents, including immunotherapy. While offering benefits to many patients, these new therapies cause significant morbidity and, in some cases, treatment-related deaths. Furthermore, clinical trials establishing these new standards of care have selective eligibility criteria that do not reflect real-world patient populations. This makes interpretation of the data and risk particularly challenging for older patients (over 65 years), patients with ethnicity other than Caucasian, and patients with comorbidities and poor performance status. In addition, the provision of cancer care is becoming more expensive, and this has important implications in terms of providing equitable access to cancer care across all Canadian jurisdictions. Estimates of the cost of treating gynecologic cancers was ~CAD 897 M in 2024, with direct health system costs projected to increase by 24% over the next decade. These projections do not account for expected advances in diagnosis and treatment. They also do not account for out-of-pocket expenses impacting patients and caregivers directly [3]. Research to discover and optimize treatments that improve survival and enhance quality of life is very important, as is research to improve access and reduce physical and financial toxicity.

The Society of Gynecologic Oncology of Canada (GOC) group is a multidisciplinary organization with a mission to improve the care of women with or at risk of gynecologic cancer. The GOC, in partnership with patients and patient advocacy groups, such as Ovarian Cancer Canada (OCC), have recognized the importance of facilitating research that bridges gaps in knowledge between trial-based evidence, real-world clinical practice and healthcare policy. Most importantly, GOC recognizes the need to conduct and encourage research that addresses important questions for patients and clinicians. 

Given the increasing Canadian interest in performing patient-centred research, with grant availability to support pragmatic clinical trials (PCTs) now becoming more commonplace, the GOC made the decision to formally launch a programme to stimulate pragmatic research. The 2024 GOC Pragmatic Clinical Trials Workshop was planned to bring together stakeholders involved in gynecologic cancers interested in PCT research and provide access to experts in the field. The meeting was held across two days in Toronto in November 2024, with patients, clinicians and scientists from across Canada (Supplementary Materials). Topics of discussion included an overview of pragmatism, methodological considerations for surgery and systemic therapy PCTs, and an overview of trial outcomes that matter as described and defined by patients. There was a break-out session to explore practical ways to address patient-prescribed research priorities. The meeting finished with interactive round table discussions with the specific objective to develop investigator-initiated pragmatic trials in gynecologic oncology surgery and systemic therapy. The goal of the meeting was to establish relationships needed to initiate coordinated and supported PCTs in gynecologic oncology, leveraging the mentorship and resources of REaCT (ReThinking Clinical Trials), IMPACTS (Innovative Multicentre Patient-centered Approach to Clinical Trials in Surgery), and Common Sense Oncology and Ovarian Cancer Canada to improve the quality of gynecologic cancer care nationally.

## 2. Meeting Preparation and Engagement Strategy

**Figure 1:**
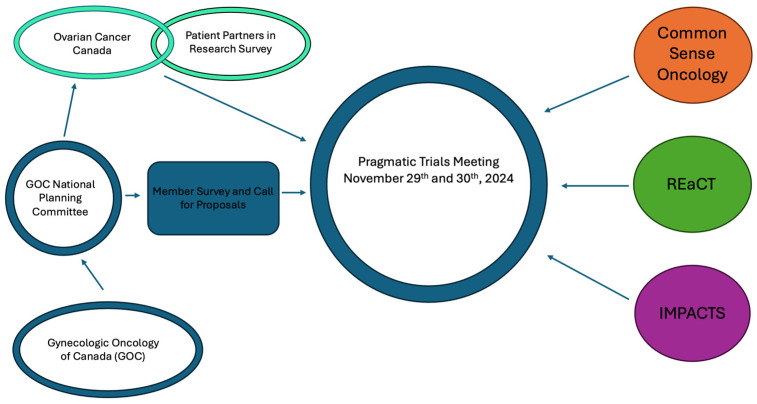
Process to develop the pragmatic trial meeting.

The GOC convened a national planning committee in March 2024 to introduce pragmatic trials to the specialty. A strategy to select participants, based on a survey and self-stated personal goals for the workshop, was developed and distributed electronically to all GOC members. To have tangible pragmatic trial concepts for discussion with the participants and invited experts, a call for a brief PCT proposal was made to the GOC members. At the same time, OCC sent out a one-page, bilingual overview of pragmatic clinical trials and a request for feedback to the national patient community through email and the online patient platform OVdialogue (Ovarian dialog) (English version: https://ovdialogue.ovariancanada.org/home) (accessed on 26 February 2025). See Figure 1. 

## 3. Workshop Overview

### 3.1. Pragmatic Trials

Invited speaker Professor Ian Tannock provided a foundation for pragmatism in clinical medicine. Pragmatic trials are designed to efficiently answer everyday, clinical questions using minimal (if any) additional healthcare resources. Pragmatic trial methodology offers broad patient inclusion, streamlined consent procedures and minimal data element collection. Pragmatic trials are also referred to as effectiveness trials, with results that are more likely to be reproducible in the ‘real-world’ patients that we treat everyday. The kinds of questions that are asked in pragmatic trials are not typically addressed through industry-sponsored randomized controlled trials (RCTs) due to the absence of potential for commercial impact and/or expansion of regulatory cancer drug approval. The design and execution of pragmatic trials date back to the 1960s, but for most practicing clinical oncologists, pragmatism is a new concept. Government grants supported 60% of RCTs in the 1970s and 1980s, but since then, clinical trials have been largely taken over by industry [4]. Industry organized and funded ~90% of phase 3 RCTs between 2010 and 2020 [5]. Industry-funded trials are conducted under near-ideal circumstances with inclusion criteria favouring patients with better performance status, fewer comorbidities and no competing risks. They are very costly to run, resource- and time-intense and require numerous extra visits, tests and procedures. These so-called efficacy trials have led to substantial gains for highly selected patients but have also led to expansive approvals for drugs that often lead to minimal, if any, improvements in outcomes in the real-world patient population [6,7]. Furthermore, many trials of targeted therapies have included a wide group of patients rather than selecting patients according to the presence of an appropriate biomarker; an example is a trial of niraparib for response maintenance in women with ovarian cancer [8]. Approvals of targeted therapies for unselected patients contradict the principle of precision medicine, which aims to match the right treatment to the right patient. In addition, the side effects of investigational drugs are often poorly assessed in industry-sponsored trials, with failure to capture chronic toxicities that lead to important changes in quality of life and are of importance to patients. The widely used CTCAE (Common Terminology Criteria for Adverse Events) criteria were developed to assess acute toxicities of conventional chemotherapy drugs and may not adequately reflect the spectrum of toxicities arising from newer targeted agents. 

Pragmatic trials are needed to compare variable standards of care in practice across sites and provinces with many oncology drug options and to evaluate the importance of the efficacy–effectiveness gap (EEG). The EEG is the difference between the results obtained in clinical trials that lead to the registration of new treatments and the results obtained in everyday practice [9,10]. Many examples of the EEG challenge confidence in decisions for drug approvals based on industry-funded RCTs, which do not take into consideration the dichotomy between ‘ideal’ versus ‘real-world’ characteristics of health systems and patients. The EEG means that the benefits are usually smaller and the toxicities are higher in a real-world clinical oncology practice when compared to industry-funded RCTs. 

### 3.2. Common Sense Oncology

Dr. Chris Booth from Common Sense Oncology (CSO) gave a presentation on how this organization aligns with and promotes PCTs in oncology. CSO is a grass-roots collective launched in 2023 that includes clinicians, academics, patients, advocates, and other stakeholders from health systems around the world. The core mission of CSO is to ensure that cancer care and innovation are focused on outcomes that matter to patients rather than the commercial bottom line. CSO’s vision is that, irrespective of where someone lives, they have access to cancer treatments that make a real difference in their lives. CSO is working with clinicians, trialists and journals to re-calibrate how RCTs are designed and reported. Dr. Booth emphasized that, under most circumstances, the primary study endpoint should be overall survival (OS) and/or quality of life (QoL). A historical perspective on how Progression-Free Survival (PFS) has largely replaced OS in terms of industry-sponsored trial endpoints was presented [5]. Dr. Booth highlighted that PFS was originally developed as a screening tool to identify signals of activity in early drug development. The limitations of using OS as a trial endpoint include longer follow-up, higher costs and control for the impact of post-progression therapies; as such, there was a drive to use PFS as a regulator-recognized endpoint. Unfortunately, PFS is not a valid surrogate for QoL or OS in most contexts, although there are some rare exceptions to this rule [11,12,13,14]. Dr. Booth emphasized that another way for clinicians to think about PFS is as ‘time to changes on a CT scan’, which may not relate to time to symptoms nor time to reinstitution of therapy. He also presented the concept of informative censoring, which means that, in practice, at least a proportion of what we see in terms of PFS benefit from randomized trials is a statistical artefact [15,16,17,18]. It is important to understand the circumstances in which we should use PFS as a trial endpoint and when we should not. As a minimum, if PFS is a primary endpoint, trials should also measure OS and QoL. CSO is working to improve how trials are designed; work streams are also underway to improve oncologist education, communication with patients, and health equity and access to cancer care. Common Sense Oncology is committed to promoting interventions that measurably improve the life of patients, celebrating well-conducted trials and challenging interventions that may cause more harm than good.

### 3.3. Rethinking Clinical Trials (REaCT)

Dr. Mark Clemons spoke about his work as co-founder of REaCT. Formed in 2014, REaCT is the world’s largest cancer pragmatic trial group in Canada. REaCT has activated over 26 oncology trials and enrolled over 5000 patients across four provinces. Dr. Clemons provided an overview of their programme, discussing some of the barriers to carrying out PCTs and some of the innovative strategies REaCT have utilized to overcome these barriers [19]. A key challenge was simplifying the patient consent process in a way that met the Research Ethics Board’s (REB) standards. The Integrated Patient Consent (IPC) model is a verbal consent process that allows the regular ‘circle of care’ staff to explain the trial during usual patient care with minimal reliance on research staff [20]. Verbal consent is documented in clinical notes with a brief information handout provided to the patient. REaCT has actively and successfully engaged with REBs in four provinces, such that a streamlined approval process for PCT protocols has been developed that does not necessarily involve a full board review at REaCT sites. 

REaCT has shown particular interest in dose optimization PCTs. Historically, the selection of dose for new agents alone or in combination has been based on small phase I dose-escalation studies that identify the maximum tolerated dose of a drug—not necessarily the maximum effective dose of a drug. The limitations of this approach have included the lack of diversity (age, comorbidity, race) of patients traditionally enrolled in phase I trials when establishing the recommended dose for further investigation. As a result, dosing based on the MTD and the recommended phase II dose (the dose level below the MTD) from phase I trials runs the risk of exposing patients to higher and potentially more toxic doses of drug than they require for the biologic effect. Furthermore, the duration of treatment for patients investigated in trials that later become adopted as a standard of care is chosen in an arbitrary manner. Hence, there is a possibility that a lower (optimized) dose or shorter duration of treatment could result in the same level of therapeutic benefit with lower toxicity (and cost). This is particularly relevant in the era of targeted and immune therapies where the mechanism of action differs substantially from traditional cytotoxic agents. Immune checkpoint inhibitors, for example, may fully inhibit their targets at lower than the current recommended doses, and the duration of inhibition may exceed the current standard of care scheduling interval. Anecdotally, patients often tell us that, “the treatment made me feel worse than the cancer”. Understanding, supporting and conducting dose optimization studies are key if we are to personalize oncology care. There are many examples, most recently with the introduction of Lenvatinib combined with Pembrolizumab in advanced endometrial cancer, where despite published trial data and regulator approved dose, practitioners are starting patients at a lower dose of the drug, based on the known toxicity and risks to patients. Investigating in a systematic manner what the optimal dose for all patients actually is, in the context of the disconnect between the science, trial data and clinical experience, is extremely important. It is essential to involve patients and oncologists in the planning of dose optimization trials, to use meaningful endpoints such as OS and QoL and to keep the trials simple [21,22,23]. 

The first part of the REaCT process is to generate PCT ideas from surveys, asking patients and oncologists for their feedback on subjects affecting care across the spectrum from diagnosis to end of life. These surveys identify variations in practice that warrant further investigation with a PCT. Before embarking on a new PCT, the REaCT team ensures that a trial addressing a similar question has not been previously reported by performing rigorous systematic reviews of the literature [19]. Given publication bias towards reporting positive trials, it is necessary to discover any meeting reports or inclusion of data in other formats which describe a negative study outcome, as sometimes practice-changing evidence is not apparent. 

A key principle highlighted by Dr. Clemons and our other experts was that PCTs should have a limited number of endpoints, tests and interventions should follow the standard of care and data collection should be sufficient to address the question but be kept to a minimum. Unlike efficacy trials, PCTs of effectiveness developed through REaCT tend to become simpler with each iteration. 

### 3.4. Innovative Multicentre Patient-Centered Approach to Clinical Trials in Surgery (IMPACTS)

Dr. Paul Karanicolas established IMPACTS, a pragmatic surgery trial programme, in 2017 to address uncertainty and poor outcomes in surgical and perioperative care. IMPACTS has several open randomized surgical oncology protocols within multiple sites in Ontario and Manitoba. He presented the programme overview, sharing several examples of surgical pragmatic trial concepts (CLEAN wound—open, three-arm randomized trial for no wound irrigation, saline wound irrigation or poviodine wound irrigation to prevent surgical site infections) that will enhance perioperative care for all patients. The IMPACTS programme searches for interventions where there is clinical equipoise in terms of benefit to patients. Historically, from the time at which an evidence gap is identified in surgical practice, it can take 10 years to address and resolve using conventional (efficacy) trial designs. IMPACTS is able to design PCTs that address practical surgical questions much faster. IMPACTS builds the infrastructure (using lessons learned from REaCT, including the IPC model) to activate trials within a few months and completes them within a couple of years so that surgeons can continually answer patient-centred, important questions. An innovative design methodology has been developed. Umbrella protocols with all standard elements included and no specific intervention listed are pre-approved by the REB and have been approved through CTO—Clinical Trials Ontario—so that once they are open at one site, they are open provincially. Platform trial and pragmatic trial methodologies (with no fixed sample size or time frame) are common. Funding agencies (i.e., CIHR—Canadian Institutes for Healthcare Research) are starting to understand these methodologies but funding still remains a challenge. IMPACTS trials try to minimize data collection using patient-reported outcomes (PROs) as much as possible, linking with other databases (to facilitate data acquisition), including NSQIP (National Surgical Quality Improvement Program) and ICES (Institute for Clinical Evaluative Science). All IMPACTS trials utilize integrated consent: clinicians provide patients with a one-page infographic, using a picture of what will happen in the trial, rather than a description in words. Patients are at the centre of IMPACTS trials, with potential trials being presented to patient groups for discussion and modification of the question and trial methodology. 

### 3.5. Putting Patients First; Advocacy and Partnership in PCTs

Dr. Alicia Tone from Ovarian Cancer Canada (OCC) together with OCC’s Patient Partners in Research (PPiR)—Karen Bemister, Shannon Kadar and Julie Mulligan—presented at the workshop. Results from an OCC survey requesting patient feedback on priorities for pragmatic clinical trials (N = 27 responses) were presented. Open-text responses were classified based on overall theme (one dominant theme per response). The most common theme was a desire for trials focused on ‘patients like me’ (26%); for instance, rare types of ovarian cancer, patients 65 years and older and individualized dosing or patterns of response by heritage/ethnicity. Other common themes included treatment side effects (22%, including impact on QoL and medical menopause), monitoring for recurrence (15%), lifestyle factors (11%), communication (11%) and consideration of comorbidities/holistic approach to care (11%). 

The PPiR representatives spoke individually to the audience, sharing their stories, perspectives, hopes and expectations from oncology care. The common thread amongst them was a desire for effective care that preserves and optimizes quality of life. To live longer but with toxicity limiting quality was not an expressed goal of care by anyone. Respect for an individualized precision approach to informed patient decision making and individualized and economically responsible drug dosing supported by a common-sense framework were specific and prominent themes. The patient partners emphasized the need for oncologists to simply ask about—rather than assume—what is important to them in terms of QoL. The assessment of side effects of therapy, for example, has been studied extensively, and there are several publications demonstrating that healthcare providers are very poor at estimating the impact and magnitude of treatment side effects on patients [24,25,26,27,28,29]. Rotating round table sessions allowed patient partners and advocates to speak individually with experts, with a focus on how to incorporate the patient voice as a central consideration in pragmatic trial design. Some specific examples of these questions, which all had a precision medicine focus, included ‘Do targeted therapies need to be dosed and scheduled in the same way for all stages of cancer or can these be modified?’, ‘Are there differences in effectiveness of therapy according to ethnic background of the patient?’, and ‘Can the activity of a PARPi (Poly—ADP Ribose Polymerase inhibitors) be enhanced by combination with any other drugs such as bevacizumab?’.

### 3.6. The Future of Pragmatic Trials

Dr. Marie-France Savard from REaCT presented future directions for PCT research in Canada with emphasis on knowledge translation and the need to leverage the energy of the entire oncologic community to ensure pragmatic trial results are disseminated widely to help improve clinical impact. She highlighted the need for early engagement during study development with local, national and international oncology organizations (such as the GOC, Choosing Wisely Canada, Optimal Cancer Care Alliance, Canadian Association of Medical Oncology, Health Canada, Federal Drug Administration, REFINE—REduced-Frequency ImmuNE checkpoint inhibition in cancer trial investigators), and patient advocacy organizations (such as OCC) to ensure that study findings are integrated into clinical practice guidelines (CPGs). To date, there are currently no practice guidelines that incorporate results from PCTs; this is a limitation affecting the applicability of CPGs to daily clinical practice. Choosing Wisely Canada could be a vehicle to disseminate PCT results to a receptive audience that is enthusiastic and supportive of practical and patient-centric treatment recommendations. This was suggested as an opportunity for GOC to take a leadership role and be the first professional organization to support the results of PCTs in this way. Dr. Savard has lobbied for funding from public institutions (in the context of publicly funded healthcare) to support dose optimization trials, given their potential cost savings for cancer care programmes across Canada. In the context of standardizing the future development of REaCT trials, a PCT review checklist has recently been implemented by REaCT. The inclusion of various criteria is based on the priority to help guarantee the success of PCTs. In part, this checklist was also necessary in helping to decide which PCT concepts to move forward with, since support for the REaCT trial infrastructure is limited. See Table 1.

REaCT trials, going forward, are committed to achieving most of the features listed in Table 1. Analysis of pragmatic trial results also needs to be pragmatic, matching the overall trial design. Based on this concept, a composite benefit–risk endpoint for patient outcomes was presented. The trial data were then summarized per intervention, and lastly, the interventions were compared [30]. This contrasts with the traditional approach, where efficacy and safety are analyzed separately and then we combine these as separate outcomes of benefit and risk. Benefit that is evaluated in isolation of risk (and vice versa) is not a patient-centric way of presenting or even thinking about therapy. Patients and clinicians want to support therapy that is effective and has the fewest number of adverse events. 

### 3.7. Pragmatic Trial Design Workshop

The final part of the GOC Pragmatic Trials Meeting was centred around developing the submitted pragmatic trial concepts. Sixteen proposals were received (six ovarian, four endometrial, two vulvar, two cervical, and two supportive care) spanning pre-invasive cancer, surgery, systemic therapy treatments and molecular monitoring of cancer. All trial concepts were reviewed by the committee and experts; the Pragmatic Explanatory Continuum Indicator (PRECIS) instrument served as a feasibility and quality guide [31]. These were sent to the expert panel in advance, and each of the experts chose a trial concept to develop during the meeting. Oncologists from across Canada were invited to participate, with representation from seven provinces.

Round table discussions were held for four projects, including (1) a dose optimization, precision oncology trial proposal for relapsed endometrial cancer patients; (2) a surgery trial in ovarian cancer; (3) a drug and dose optimization trial in platinum-resistant ovarian cancer; and (4) an intervention trial of geriatric assessment in optimizing precision immune-oncology therapy for older patients with metastatic endometrial cancer. A fifth table focused on working through the patient survey results presented earlier in the meeting, with the goal of identifying which ideas could be answered using a pragmatic approach. 

For some proposals, there was progression to some of the more intricate details relating to the trial interventions (i.e., what drug doses to compare), and in others, larger issues were discussed, such as how to guarantee sufficient enrollment of patients to evaluate the trial’s primary outcome. Each group reported to the larger group at the end of the session with additional suggestions made for these trial concepts. Future steps and a group consensus are summarized in Table 2.

## 4. Discussion

A follow-up meeting to debrief was held two weeks after the symposium with the REaCT team to explore collaborative opportunities and elaborate on some of the identified future steps. Operational models based on individual participant trial centres serving as the sponsor but utilizing REaCT expertise were discussed. The GOC is moving forward with plans to develop PCTs and raise their profile further for patients with gynecologic cancers. 

Shortly following the meeting—and in response to calls for more inclusive and equitable clinical trials from the patient community—Ovarian Cancer Canada launched an open competition focused on pragmatic trial protocols. BioCanRx (Canada’s Immunotherapy Network) joined as a 50:50 partner following the great success of the meeting, bringing the total funds available to Canadian researchers to CAD 800,000. It is anticipated and hoped that discussions and lessons from the meeting will result in several applications being submitted for consideration of funding. 

We are also witnessing a resurgence of interest in pragmatic trials among oncologists globally. The Gynecologic Cancer InterGroup (GCIG) have scheduled a Brainstorming Meeting for 2025. In January 2025, the NRG (name derived from the three parental groups—National Surgical Adjuvant Breast and Bowel Project, the Radiation Therapy Oncology Group and the Gynecologic Oncology Group) conducted its first pragmatic trial symposium. 

## 5. Conclusions

As a national society, the GOC has the opportunity to develop PCTs by providing structure, mentorship and a national framework to help activate pragmatic trial protocols. The GOC can provide a forum and venue for the translation and presentation of PCT results. Collaboration with REaCT, IMPACTS and Common Sense Oncology will ensure that we can be successful in scaling-up and reaching across disease sites in a coordinated strategy to make PCT results available and applicable to the global cancer patient community. Most importantly, partnership with patients and patient advocacy groups, including Ovarian Cancer Canada, will be essential to design relevant studies that our patients want and need. Finally, the GOC, together with patients and our partner organizations, can advocate for funding and the incorporation of PCT outcomes with CPGs to facilitate practice change at provincial and national levels. We have the knowledge, interest and support we need to improve cancer care in real time with engaged clinicians working directly with their patients. It is up to us to get it done and the time is now.

## Figures and Tables

**Table 1:** REaCT trial checklist.

Trial Attribute	Description
Principal Investigator (PI)	- Must attend REaCT weekly meetings to receive and give updates on accrual; problem solving opportunity with team
Trial Design	- Must be pragmatic - Must utilize integrative consent model - Few eligibility criteria;
Trial Resources	- No additional visits or tests beyond standard of care - No reliance on outside services to accomplish trial activities (e.g., radiology or other outside hospital service)
Trial Data Collection	- Must leverage the Electronic Medical Record - Must use patient portal to collect patient-reported outcomes
Trial Site(s)	- Limited to committed sites where site PIs are champions for the trial. Primary site PI should be able to accrue at least 60% of all trial participants alone.

**Table 2:** Future steps toward pragmatic trial expansion within the GOC.

Recommendation	Action
Secure independent funding	GOC Business Group and Research Unit to explore sponsorship opportunities and work closely with OCC to develop pragmatic trial questions with patient direction and support, thereby leveraging OCC grant opportunities
Collaboration with REaCT	A GOC investigator REaCT collaboration was established. REaCT were eager to share their trial protocols and procedures and to open REaCT sites so the GOC may benefit from work already performed to open and support pragmatic trials at these sites
Collaboration with Choosing Wisely	GOC and REaCT contact Choosing Wisely and explore interest in publication of pragmatic trial outcomes to impact patient care nationally and internationally
Collaboration with Common Sense Oncology	GOC members will be encouraged to become members and to keep informed of advocacy work by CSO
Collaboration with IMPACTS	GOC members have already expressed interest to open IMPACTS trials at their home sites where benefits apply to all patients undergoing surgical procedures
Education (knowledge translation)	The participants recognized that the PCTs are of interest to learners and the broader GOC community. Communication with GOC leadership to hold an education session for trainees at the Continuing Professional Development (CPD) meeting and incorporate RCT and PCT findings as part of GOC events.

## Supplementary Materials

The following supporting information can be downloaded at: https://www.mdpi.com/article/10.3390/curroncol32030139/s1.

## Abbreviations

The following abbreviations are used in this manuscript:

CTCAE	Common Terminology Criteria for Adverse Events
GCIG	Gynecologic Cancer InterGroup
GOC	Society of Gynecologic Oncology of Canada
EEG	Efficacy Effectiveness Gap
ICES	Institute for Clinical Evaluative Sciences
IMPACTS	Innovative Multicentre Patient-Centered Approach to Clinical Trials in Surgery
IPC	Integrated Patient Consent
MTD	Maximum Tolerated Dose
M	Million
NRG	National Surgical Adjuvant Breast and Bowel Project, the Radiation Therapy Oncology Group and the Gynecologic Oncology Group
OS	Overall Survival
OV	Ovarian
PARPi	Poly-ADP ribose polymerase inhibitor
PRO	Patient-Reported Outcome
PI	Principal Investigator
PFS	Progression-Free Survival
QoL	Quality of Life
RCT	Randomized Controlled Trial
REaCT	REthinking Clinical Trials
PCT	Pragmatic Clinical Trial
