# Supplementary material for: Putting Patients First: Pragmatic Trials in Gynecologic Oncology
Source: Curr Oncol. 2025 Feb 27;32(3):139. doi: 10.3390/curroncol32030139 (PMC11941110; doi:10.3390/curroncol32030139)
Supplement: Supplementary file 1 [file curroncol-32-00139-s001.zip › PCT_Nov30.pdf]

Clinical Trials Agenda: Workshop
November 30th, 2024

8:00am Welcome and Speakers Introductions - Helen MacKay

8:15am Common Sense Oncology; Outcomes that Matter to Patient - Chris Booth

8:55am The Rethinking Clinical Trials Program – a pragmatic trials program for patients - Mark Clemmons

9:35am Transforming Clinical Trials into Clinical Care – the IMPACTS program - Paul Karanicolas

10:15am **BREAK**

10:45am Understanding Patient-Centered Values - Alicia Tone

11:05am Round Tables (20 minutes each):

- Table 1: Patient focused research outcomes - *Chris Booth & Mary France*
- Table 2: Pragmatic Trials quality assessment - *Ian Tannock*
- Table 3: Pragmatic Trials – Creating a pragmatic trials platform - the good, the bad and the ugly - *Mark Clemons*
- Table 4: Picking questions that matter to patients *Paul Karanicolas*

Rotations at: 11:25; 11:45; & 12:05

12:25pm **LUNCH AND NETWORKING**

Clinical Trials Agenda: Workshop

November 30th, 2024

13:10am The Next Frontier of Pragmatic Trials in Canada: All Hands on Deck! - *Marie-France Savard*

13:50pm Break-out Groups

15:00pm Report Session Closeout Group present a report on the discussion, next steps, etc

16:00pm Final Remarks: Grants announcement/next steps/program evaluation - Helen MacKay
