# Supplementary figures and images for: Putting Patients First: Pragmatic Trials in Gynecologic Oncology
Source: Curr Oncol. 2025 Feb 27;32(3):139. doi: 10.3390/curroncol32030139 (PMC11941110; doi:10.3390/curroncol32030139)

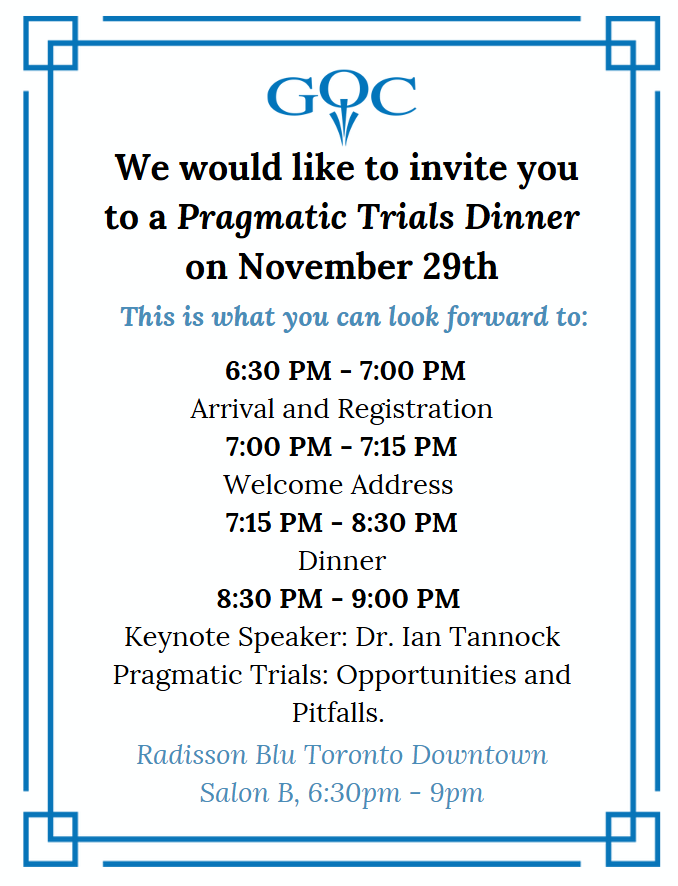

Supplement: Supplementary file 1 [file curroncol-32-00139-s001.zip › PCT_Nov 29.png]
